# FFPE breast tumour blocks provide reliable sources of both germline and malignant DNA for investigation of genetic determinants of individual tumour responses to treatment

**DOI:** 10.1007/s10549-018-4798-7

**Published:** 2018-04-26

**Authors:** Anna Wilkins, Ritika Chauhan, Alistair Rust, Alex Pearson, Frances Daley, Floriana Manodoro, Kerry Fenwick, Judith Bliss, John Yarnold, Navita Somaiah

**Affiliations:** 1 Division of Radiotherapy and Imaging, The Institute of Cancer Research, London, UK; 2 The Institute of Cancer Research Clinical Trials and Statistics Unit, London, UK; 3 Tumour Profiling Unit, The Institute of Cancer Research, London, UK; 4 The Royal Marsden Hospital, Downs Road, Sutton, SM2 5PT UK; 5 Division of Breast Cancer Research, The Institute of Cancer Research, London, UK

**Keywords:** Germline determinants, Whole exome sequencing, Breast cancer, Archival formalin fixed paraffin embedded tissue

## Abstract

**Background:**

Bio-banked formalin-fixed paraffin-embedded (FFPE) tissues provide an excellent opportunity for translational genomic research. Historically matched blood has not always been collected as a source of germline DNA. This project aimed to establish if normal FFPE breast tissue could be used as an alternative to blood.

**Methods:**

Exome sequencing was carried out on matched tumour tissue, normal breast tissue and blood on five patients in the START trial. Retrieved samples had been archived at different centres for at least 13 years. Following tissue macro-dissection and DNA extraction, targeted exome capture was performed using SureSelect Human All Exome v5 reagents (Agilent). Illumina paired-end libraries were prepared from the captured target regions and sequenced on a HiSeq2500 (Illumina) acquiring 2 × 75 bp reads. Somatic variants were called using the MuTect software analysis tool and copy number abnormalities (CNA) were identified using CNVkit. Targeted sequencing and droplet digital PCR were used to validate somatic variants and CNA, respectively.

**Results:**

Overlap of somatic variants and CNA called on tumour versus blood and tumour versus normal breast tissue was good. Agreement in somatic variant calling ranged from 76.9 to 93.6%. Variants with an allele frequency lower than 10% were more difficult to validate irrespective of the type of germline DNA used. Pearson’s correlation coefficients for paired comparisons of CNA using blood or normal tissue as reference ranged from 0.70 to 0.94.

**Conclusions:**

There is good correlation between the somatic mutations and CNA called using archived blood or normal breast tissue as germline reference material.

**Electronic supplementary material:**

The online version of this article (10.1007/s10549-018-4798-7) contains supplementary material, which is available to authorised users.

## Introduction

Bio-banked formalin-fixed and paraffin-embedded (FFPE) tissues provide an excellent opportunity for translational research. Reductions in cost and the wider availability of next-generation sequencing (NGS) in the research community has meant that this sequence-level analysis is increasingly being used in cancer genetics. For DNA studies, matched germline blood is typically used for exome and whole genome sequencing to provide a “normal” reference against which an individual patient’s abnormal tumour DNA can be compared. Historical clinical sample collection, prior to the widespread availability of NGS, did not usually include collection of germline blood. An important pragmatic question, therefore, arises as to whether surrounding normal tissue can be used as an alternative reference germline from these archival samples. This proof-of-principle study was designed to assess the feasibility and validity of using normal breast tissue as a reference germline in a small cohort of patients where all three of normal breast tissue, breast tumours and blood samples were available in a historical cohort.

“Field cancerisation” describes areas around tumours consisting of histologically normal, yet genetically aberrant cells, and is well-recognised in breast cancer [1]. Genomic alterations including allelic imbalance and loss of heterozygosity (LOH) [2], aneusomy [3] and dysfunctional telomeres [4] have been demonstrated in histopathologically normal breast lobules, and the frequency of some aberrations has shown a correlation with distance from the tumour [5]. Such changes typically occur in only a proportion of epithelial cells and are unlikely to be present in other cell types present in normal breast tissue. Other cell types include adipocytes, stromal, endothelial and smooth muscle cells, and possibly cells associated with inflammation or the immune response. The impact of field cancerisation may therefore be minimised by selection of normal breast tissue located at a distance from the tumour, which will typically include a mixture of epithelial and non-epithelial cells.

A previous study used polymerase chain reaction (PCR) to assess five genotypes with frequent LOH in breast cancer and demonstrated 100% concordance for genotyping from FFPE normal tissue adjacent to tumour and from blood [6]. The International cancer genome consortium (ICGC) conducted a large sequencing study to assess the landscape of somatic mutations in 560 breast cancer whole-genome sequences [7]. For 96 patients adjacent breast tissue was used as the source of normal DNA, whereas for 353 patients blood was used as the source of germline DNA. Although a formal comparison between the sources of germline DNA was not reported, no significant differences in sequencing findings were noted in cases using adjacent breast versus those using blood for germline DNA.

The cancer genome atlas (TCGA) recently conducted a multi-platform analysis using fresh frozen samples. DNA defects, epigenetic changes and gene expression profiles were reported from cancer-adjacent breast tissue, defined as at least 2 cm from breast tumour [8]. Although changes consistent with field cancerisation were identified, these did not appear to prohibit use of normal breast tissue as the germline reference. Sequence data from tumours and cancer-adjacent breast tissue were each compared with blood, and therefore the genomic comparisons conducted in the TCGA analysis were not the same as our study.

The work in this study aimed to establish if DNA extracted from archived normal breast tissue blocks can be used as a surrogate germline reference by comparing exome sequencing of breast tumours using either matched blood DNA or normal breast DNA as the germline reference.

## Methods

### Selection of patient samples

Five patients with contemporaneous plasma and breast samples were selected from different centres participating in the START radiotherapy fractionation trial (ISRCTN59368779), so as to represent a cross section of the larger trial cohort. Matched archival FFPE blocks for tumour tissue and normal breast tissue plus frozen blood samples for these patients were identified. The samples had been stored for at least 14 years. Normal breast tissue blocks were chosen that were considered as free as possible of tumour cell contamination or pre-malignant change, following review of Haematoxylin and Eosin (H & E) sections by a specialist breast cancer pathologist. This means that individual sections flanking the H & E section were highly likely to be free of malignant or pre-malignant change. Eligibility criteria for the START trial included complete macroscopic excision of tumour by breast-conserving surgery or mastectomy (ideally, no microscopic evidence of invasive or in situ cancer at, or within 1 mm of, a resection margin). Three patients had undergone lumpectomy plus wide local excision and two patients had undergone mastectomy. It was not possible to retrospectively quantify the exact distance from tumour but the existence of tumour-free blocks suggests the margins from invasive tumour were generous.

### DNA extraction

Sections (3 × 8 µm) were cut from tissue blocks, and stained with Nuclear Fast Red to guide macro-dissection. Tumour areas were macro-dissected following demarcation of malignant tissue on an H & E slide by a specialist breast pathologist, with the aim of maximising tumour purity. Normal breast tissue blocks did not undergo any macro or micro-dissection. Tumour and normal breast DNA was extracted using the QIamp DNA FFPE Tissue Kit (Qiagen) as per the protocol instructions. DNA was eluted in approximately 70 µl of ATE buffer and stored at − 20 °C. DNA from blood was extracted using the Qiagen Blood and Tissue kit following the manufacturer’s instructions. DNA was quantified using Qubit fluorometry.

### Exome sequencing and data analysis

Targeted exome capture was performed using SureSelect Human All Exome v5 reagents (Agilent). Illumina paired-end libraries were prepared from the captured target regions and sequenced on a HiSeq2500 (Illumina) using v4 chemistry acquiring 2 × 75 bp reads.

BWA-mem (v0.7.5a) was used to align reads to the human reference genome (GRCh37). PCR duplicates were removed prior to further processing and variant detection. Somatic variant calling on tumour samples paired with matched normal samples was performed to identify both single nucleotide variants (SNVs) and small insertions/deletions (indels) of < 30 bp. Somatic SNVs were identified using MuTect (v1.1.4; http://www.broadinstitute.org/cancer/cga/mutect). Somatic indels were selected using the Genome Analysis Toolkit (GATK) v3.3.0. All somatic variants were annotated using the SnpEff [9], which provided information on genes affected by mutations and the likely consequences for the encoded gene products. Variants called in regions not covered by the capture probes were excluded, as were those with genotype quality scores below 20, alternative read count less than 10, variant allele frequencies (VAFs) less than 2% and coverage less than 20 reads in either sample.

CNVKit v0.8.5 [10] was used to derive somatic copy number alterations (CNA) by comparing the tumour sample to the matched germline sample (normal breast tissue or blood). CNVkit calculates the mean read depths for on-target and off-target reads which are then combined for each interval. The combined mean read depths were normalised against the control sample and corrected for systematic biases (such as GC content) to obtain the log2 copy number ratios. Discrete copy number segments were generated from the bin level log2 ratio values using the circular binary segmentation (CBS) algorithm [11], which is available as one of the segmentation methods within CNVKit. The copy number values and frequency of heterozygous mutations were used for the estimation of tumour purity using the ASCAT software package (8). The observed log2 copy number ratios were adjusted using the purity estimates to generate the absolute copy number values. Adjacent segments with the same allele specific copy number were merged using the “filter cn” option in CNVkit.

Two different approaches for description of CNA were used; matched and pooled. First, individually matched tumour versus normal tissue or blood comparisons was conducted. Second, a pooled analysis using all tumour-free normal breast tissue samples or all blood samples as reference germline, compared to each individual tumour sample, was performed [12].

### Validation of somatic variants using a customised AmpliSeq panel

A customised Ampliseq panel assessing 139 somatic variants detected using blood or normal breast tissue as reference was developed using the Ion AmpliSeq Designer. This panel was designed to validate mutations with a discrepant call between normal breast and blood, and variants of particular relevance to cancer [cancer genomics consortium (CGC) genes]. In order to explore the limit of detection of variants, it also included a number of variants with a VAF on exome sequencing that was lower than 10%. As there were slightly more somatic variants detected using blood as germline versus normal breast, the Ampliseq panel was enriched for discrepant variants detected using blood as the germline (*n* = 35) rather than normal breast as the germline (*n* = 4).

Sequencing libraries were prepared using the automated Ion Chef library preparation protocol, using a single pool of 10 ng of DNA. Libraries were templated using the Ion Chef (Life Technologies), and sequenced on a PI chip using the Ion PI HiQ sequencing reagents (Life Technologies), 520 flows, with an average amplicon length of 117 bp, to a minimum depth of ×18,000. The sequencing resulted in 3,363,175–7,408,316 reads per sample. The sequencing data were processed using the Ion torrent suite v5.0.4. Reads were aligned to the human reference genome (GRCh37). Coverage data were generated using the Coverage Analysis plugin v5.2. Ion torrent Variant caller (TVC) plugin v5.2 with no Hotspot region and the configuration “Somatic Low Stringency” was used for calling variants. The variants called in the Ampliseq panel were then compared with the Illumina exome sequencing data.

### Validation of copy number abnormalities using digital polymerase chain reaction (ddPCR)

Droplet digital PCR (ddPCR) was used to validate copy number abnormalities. ddPCR was performed on a QX200 digital PCR system (Bio-Rad) using the assays described in table S1. CGC genes with the highest copy number gain (≥ 4 absolute copies) were prioritised as these are likely to be of most clinical impact and ddPCR has been shown to detect these robustly [13, 14]. Genes showing different copy number gain using either blood or normal breast as a reference were also prioritised to indicate which reference germline provided most accurate estimation of the relevant CNA. 1–2 ng of tumour tissue DNA was used per assay. Reactions were prepared using digital PCR Supermix for probes (Bio-Rad) and a QX200 droplet generator, this system produces approximately 20,000 droplets per reaction. PCR experiments were run on a 96-well plate on a G-storm GS4 thermal cycler, incubating the plates at 95° for 10 min followed by 40 cycles of 95° for 15 s and 60° for 60 s, then 10 min incubation at 98°. Plates were read on a Bio-Rad QX200 droplet reader with QuantaSoft v1.6.6.0320 software to quantify the number of droplets positive for the genes being assessed. Four reference genes were used for each test gene, together with at least two negative control wells with no DNA in every run. Positive and negative controls using cell lines with and without the relevant copy number alteration, respectively, were also included in each experiment. CNA for target genes were calculated as a ratio with the median of the four multiplexed reference genes.

## Results

### Quality of NGS

Sample description, library size, exome capture and sequencing metrics are displayed in Table 1. Overall, these indicate that the DNA libraries were of good quality and exome capture was performed robustly with more than 100X median coverage for all samples. Exome capture and coverage was slightly lower for blood samples (median depth 107X–125X) than FFPE samples (median depth 134X–167X).

**Table 1:** Sequencing quality control

Sequencing parameter	FFPE samples (*n* = 10)	Blood (*n* = 5)
Library size (bp)	236–278	324–328
Exome capture % mapped	89–94%	96%
% Duplicates	10–15%	6%
% On targets	74–78%	70–74%
Median depth	134X–167X	107X–125X

*FFPE* formalin-fixed paraffin-embedded

### Somatic variant detection

**Fig. 1:**
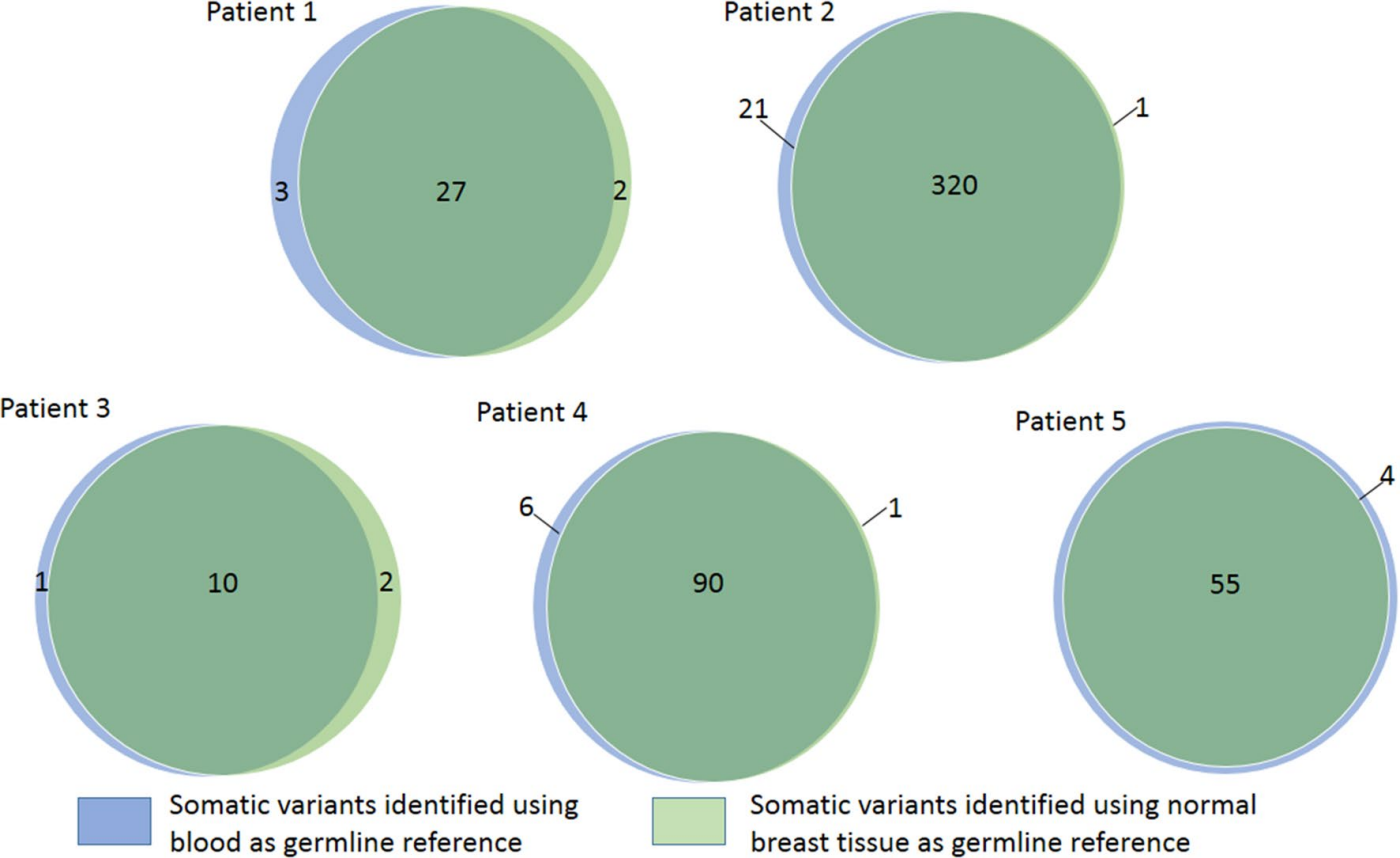
The overlap of somatic variants called with blood or normal breast tissue as germline reference

Following variant filtration, the number of somatic variants (either SNVs or small indel) called per tumour using blood as a germline reference ranged from 11 to 341 and the number of variants called using normal breast tissue as a germline reference ranged from 12 to 321. The overlap of somatic variants called on tumour versus blood and tumour versus normal breast tissue is shown in Fig. 1 and ranged from 76.9 to 93.6% (Table 2, S2). This indicates a good agreement between variants called using either germline reference. The specific somatic variants identified for each sample, and using both germline samples, are tabulated in the supplemental data. Somatic variants that are known recurrent driver mutations in breast cancer [7, 15, 16] are also tabulated, for which agreement in calling using either germline remains very good (table S3, S4, S5).

**Table 2:** The overlap of somatic variants (both SNVs and short indels) called in tumour using either blood or normal breast tissue as the germline reference using exome sequencing and targeted Ampliseq validation

Exome sequencing				
Sample no.	Total variants versus normal breast tissue	Total variants versus blood	Shared variants	Percentage of variants shared using normal breast or blood as reference (%)
1	29	30	27	84.38
2	321	341	320	93.57
3	12	11	10	76.92
4	85	90	84	92.31
5	55	59	55	93.22

Targeted Ampliseq validation				
Sample no.	No of variants with results	Variant present in exome sequencing using blood as reference	Variant present in exome sequencing using normal breast as reference	Percentage of shared exome sequencing variants validated by Ampliseq panel (%)
1	15	15	14	93.3
2	57	57	41	71.9
3	2	2	2	100.0
4	28	28	27	96.4
5	8	8	7	87.5
Total	110	110	91	

***SNV*** single nucleotide variation

Somatic variants identified using exome sequencing were validated using targeted sequencing consisting of an Ampliseq panel to test 139 selected variants (the specific variants tested are listed in the supplemental appendix, table S6). As outlined in the “Methods” section, variants with discrepant calls between each germline and variants of specific relevance to cancer were prioritised in this panel. 110/139 (79.1%) of mutations were called using the Ampliseq panel. Of the 29 mutations that were not successfully called using the Ampliseq panel, but present in the exome sequencing data, 19 occurred at VAF less than 10% in exome sequencing and 11 of these had VAF less than 5%. A further three mutations in the Ampliseq panel had lower than average coverage (< 1000 reads), and two mutations had poor mapping quality. This indicates a number of the mutations that were not successfully called had identifiable poor quality metrics.

Thirty nine of the 41 total somatic variants with a discrepant call between normal breast and blood were included in the Ampliseq panel. Of these, 35 variants were detected using blood and not normal breast, and four variants were detected using normal breast and not blood. Of the 39 discrepant variants in the Ampliseq panel, successful calls were obtained for 19 variants, all of which had been identified using blood as the germline, and not normal breast tissue. For the remaining 21 variants, there was no coverage in the relevant regions and hence it was not possible to discern mutation status. Manual review of the 19 variants suggested that for the majority of variants, the relevant base change only occurred in a small number of sequencing reads and often towards the end of reads (where read qualities decrease). In addition, the majority of changes were G to A and C to T base changes. Although it is not possible to definitively categorise these changes, the manual review indicates that both sequencing error and FFPE artefact explain a substantial proportion of these discrepancies.

Over 300 somatic variants were called with each reference germline from sample number 2. This sample may have been a hypermutated tumour arising for example from mismatch repair deficiency or APOBEC-induced mutagenesis (see supplemental data for COSMIC somatic mutation signatures, figure S1). The high total number of somatic variants called in sample 2 means that it is not surprising that 15 of the 19 (78.9%) variants with discrepant calls arose from this sample. It is possible that field cancerisation explains some of the discrepant calls in this sample; however, FFPE-induced artefact may have also increased the likelihood of discrepant calls.

### Matched analysis of CNA

**Fig. 2:**
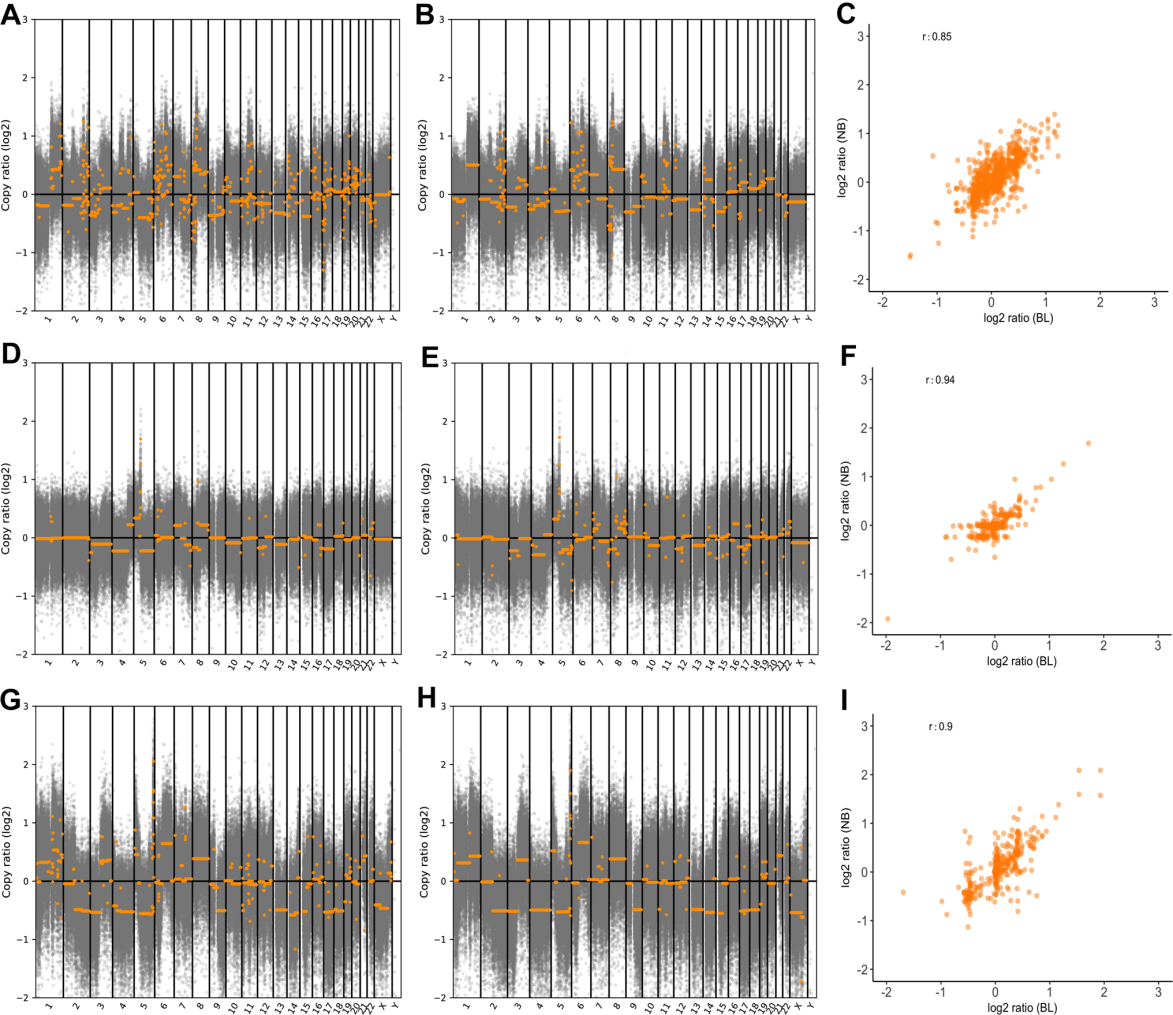
Copy number profiles using normal breast or blood as germline reference and correlation between references. **a** and **b** Copy number profiles showing CNA called using both normal breast and blood as germline, respectively, for patient 1, **c** Scatterplot showing correlation of CNA using normal breast or blood as germline patient 1. **d** and **e** Copy number profiles showing CNA called using both normal breast and blood as germline respectively for patient 4, **f** Scatterplot showing correlation of CNA using normal breast or blood as germline patient 4. **g** and **h** Copy number profiles showing CNA called using both normal breast and blood as germline respectively for patient 5, **i** Scatterplot showing correlation of CNA using normal breast or blood as germline patient 5. *CNA* copy number alteration, *BL* blood, *NB* normal breast tissue

Estimates of tumour purity by a specialist histopathologist and the ASCAT analysis software package ranged from 52 to 90% (table S7). CNA detected using either matched blood or normal breast tissue as germline material are shown as copy number profiles and scatter plots in Fig. 2 for patients 1, 4 and 5, respectively, and for patients 2 and 3 in the supplementary appendix (figure S2). Visual comparison of these plots suggests considerable variation in genomic instability between samples but overall a good correlation between CNA identified using either germline reference. Pearson’s correlation coefficient for paired comparisons of CNA using either reference ranged from 0.70 to 0.94.

Validation of CNA was carried out using ddPCR for five genes. As explained above, these genes were selected as having gains of at least four copies using at least one germline reference from the exome sequencing output. Five out of six gains (83.3%) were validated using ddPCR as shown in Table 3.

**Table 3:** ddPCR validation of CNA

Gene	Sample no.	Exome sequencing (absolute copy number)		ddPCR ratio	ddPCR result
		Tumour versus normal breast	Tumour versus blood		
MALAT1	2	3	5	2.07	Gain
	4	3	4	1.69	Gain
IKBKB	1	3	5	1.84	Gain
TERT	4	4	3	2.13	Gain
HIST1H3B	2	3	5	0.92	No gain
IRF4	1	4	3	1.54	Gain

*ddPCR* droplet digital polymerase chain reaction

### Pooled analysis of CNA

Following matched analysis of CNA, all five blood samples were combined to give a ‘pooled blood germline reference’ and all five normal breast samples were combined to give a ‘pooled normal breast reference.’ We considered this might be a useful approach to reduce sequencing noise, such as FFPE-induced artefact, from individual sample comparisons. These pooled references were then compared with individual tumour samples. Overall, these comparisons did not improve resolution of CNA and instead appeared to add artefactual noise. Copy number profiles using these pooled comparisons for patients 1 and 5 are shown in the supplementary appendix figure S3. A limitation of these pooled comparisons is that they were derived from only five patients.

## Discussion

This study sought to identify if matched normal breast tissue from FFPE blocks, free of malignant or pre-malignant change on immunohistochemistry, could provide a reliable source of germline DNA with which tumour DNA could be compared using whole exome NGS. To help establish reliability, matched blood and tumour DNA were also compared. Findings from NGS were validated with targeted sequencing using an Ampliseq panel and droplet digital PCR. We acknowledge the small sample size which limits a rigorous assessment of the impact of factors such as specimen age on outcomes. However, this was a proof-of-principle study based on patients where all three of blood, normal tissue and tumour samples were available from a historical cohort.

We were able to prepare libraries and carry out whole exome sequencing using DNA from old archival FFPE samples retrieved from different treatment centres. This was despite the effects that fixation can have on DNA including hydrolysis, deamination and DNA–protein cross-linking, all of which can compromise library production. In addition, a good correlation between genomic aberrations called with blood as reference versus those called with normal tissue from the tumour resection specimen was seen.

With the use of stringent filtration criteria, there was a very good agreement between somatic variants identified, including for known recurrent driver mutations in breast cancer [7, 15, 16], using either normal breast tissue or blood as a germline reference. Our filtration criteria were selected based on the MSK-IMPACT experience [12]. Whilst it is possible that less stringent criteria may have identified more true somatic variants (see supplementary data, table S3, S8), our concern is that more false positive variants, typically induced by FFPE artefact, would also have resulted. Overall, as expected, variants with a VAF of more than 10% were validated most consistently. Almost all variants with a discrepant call between each germline sample on exome sequence were included in the validation panel. Those discrepant calls that were successfully sequenced using the Ampliseq panel were validated as true somatic variants. It is difficult to confirm whether such discrepant calls using the different germline references relate to FFPE-induced artefact, sequencing error or field cancerisation, however, it is relevant that these discrepancies occurred infrequently and represented less than 10% of all variant calls.

It is encouraging that correlation in CNA using either reference germline was also very good across the five analysed samples. If possible, fresh frozen tissue is increasingly used for NGS, particularly whole genome or whole exome sequencing, because it avoids the artefacts associated with formalin fixation. Our results suggest it is reasonable to use FFPE tissue in this context. We recognise that a limitation of this study is that the ddPCR platform used for validation only enabled reliable assessment of copy number gains with four or more copies, and not losses, and that a fairly small number of genes were selected for validation.

We attempted a number of strategies to try and reduce noise from copy number data, including the use of pooled references and trying different segmentation and filtering approaches. Using the CBS segmentation method, along with the CNVkit “filter” option, removed a significant proportion of the spurious copy number segments; however, we did not see any significant improvement using the pooled reference approach. This could be due to the fact that our pooled comparison only used five samples. Most clinical studies with a pooled reference would involve a much larger pool of reference samples which is likely to improve reliability of CNA detection [12]. The use of online repositories of pooled reference germline DNA, e.g. from The Cancer Genome Atlas (TCGA) or International Cancer Genome Consortium (ICGC) databases could be an alternative source of germline genomic data.

The recently reported analysis by TCGA of DNA defects, epigenetics and gene expression in cancer-adjacent breast tissue [8] concluded that approximately 40% of benign-appearing breast-adjacent tissue harboured genomic defects in DNA copy number, sequence, methylation status or in RNA sequence. However, for the 40 samples that underwent exome sequencing, clear detectable CNA were rare, occurring in 10% or less of cases. In addition, although 25% of cancer-adjacent samples had moderate to high levels of tumour-like somatic mutations, the variant allele fraction (VAF) was typically low. For example, five out of six mutations seen in TP53 occurred at a VAF of less than 1%, all of these mutations would have been filtered by the stringent criteria typically used for FFPE samples and in this study. Whilst the field cancerisation changes characterised in detail in the TCGA report are important, we do not believe that they invalidate the use of FFPE normal breast DNA as a germline reference. It is important that careful histopathological review of normal breast tissue is carried out and appropriate filtering of somatic variants. We propose exclusion of somatic variants with genotype quality scores below 20, alternative read count less than 10, variant allele frequencies (VAFs) less than 2% or coverage less than 20 reads.

In summary, this study sought to establish if archival DNA extracted from histologically normal FFPE breast tissue blocks could be used as a germline reference against tumour DNA, when germline blood is not available. It is relevant that we used old archival tissue samples retrieved from different treatment centres where they had been stored for at least 14 years. Somatic variants identified using either matched blood or normal breast tissue had a very good agreement, suggesting that normal breast tissue can be used as a reliable surrogate to identify these aberrations with appropriate filtration criteria. Such filtration criteria appear to minimise the impact of both FFPE-induced artefact and any field cancerisation changes that may be present. Although CNA were more complicated to directly compare, copy number abnormality calls were consistent using either germline reference and key copy number gains were validated. Overall, this study suggests that normal breast DNA from archival FFPE blocks lacking malignant or pre-malignant change can be used as a surrogate germline and offers a robust alternative to use of DNA from blood for whole exome sequencing.

## Electronic supplementary material

Below is the link to the electronic supplementary material.

Supplementary material 1 (DOCX 1392 KB)

Supplementary material 2 (DOCX 25 KB)

Supplementary material 3 (DOCX 37 KB)

Supplementary material 4 (CSV 4 KB)

Supplementary material 5 (CSV 49 KB)

Supplementary material 6 (CSV 2 KB)

Supplementary material 7 (CSV 12 KB)

Supplementary material 8 (CSV 8 KB)

Supplementary material 9 (CSV 5 KB)

Supplementary material 10 (CSV 52 KB)

Supplementary material 11 (CSV 1 KB)

Supplementary material 12 (CSV 13 KB)

Supplementary material 13 (CSV 9 KB)
