# Supplementary material for: FFPE breast tumour blocks provide reliable sources of both germline and malignant DNA for investigation of genetic determinants of individual tumour responses to treatment
Source: Breast Cancer Res Treat. 2018 Apr 26;170(3):573–81. doi: 10.1007/s10549-018-4798-7 (PMC6022520; doi:10.1007/s10549-018-4798-7)
Supplement: Supplementary file 1 — Supplementary material 1 (DOCX 1392 KB) [file 10549_2018_4798_MOESM1_ESM.docx]

**Figure S1: Cosmic somatic mutation signatures pie plot, Alexandrow, Nature 2013**

**Figure S2: A and B:** Copy number profiles showing CNA called using both normal breast tissue and blood as germline respectively for patient 2. **C:** Scatterplot showing agreement of CNA using normal breast tissue or blood as germline for patient 2. **D and E:** Copy number profiles showing CNA called using both normal breast tissue and blood as germline respectively for patient 3, **F:** Scatterplot showing agreement of CNA using normal breast tissue or blood as germline for patient 3

**
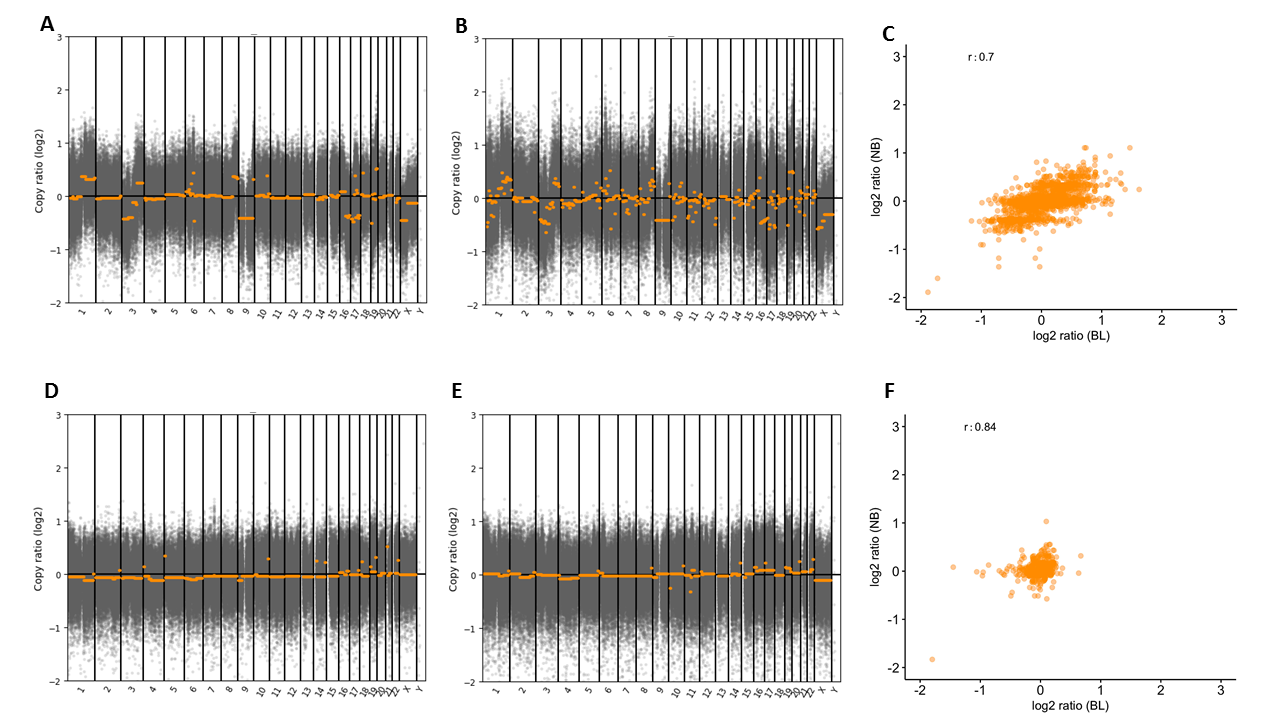
**

**Figure S3**: Copy number profiles using pooled germline normal breast tissue DNA and pooled germline blood DNA. **A:** Patient 1 Tumour versus pooled blood, **B:** Patient 1 Tumour versus pooled normal breast tissue. **C:** Scatterplot showing agreement of CNA using normal breast tissue or blood as germline for patient 1. **D:** Patient 5 Tumour versus pooled blood. **E:** Patient 5 Tumour versus pooled normal breast tissue. **F:** Scatterplot showing agreement of CNA using normal breast tissue or blood as germline for patient 5

**
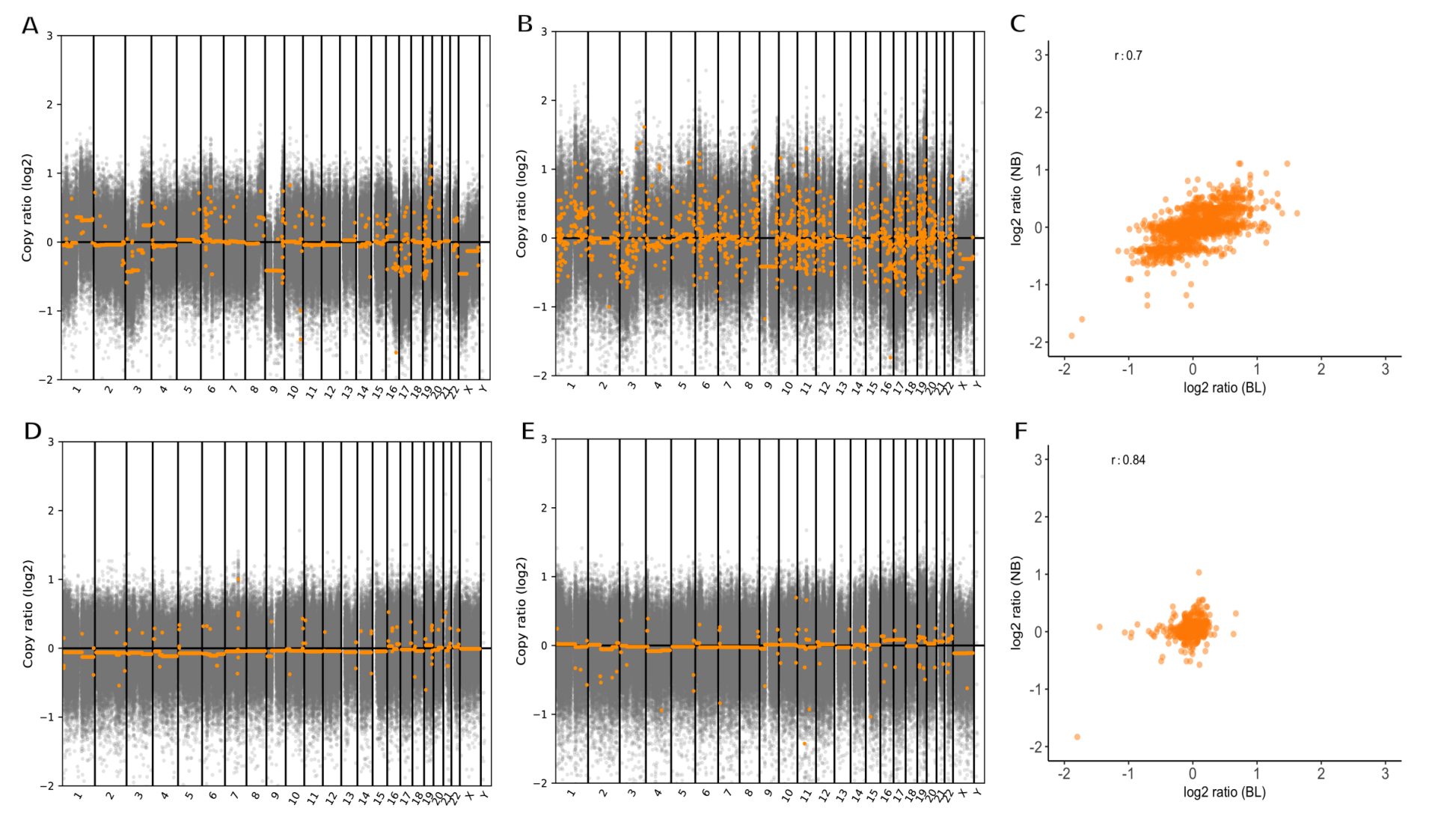
**
