# Supplementary material for: FFPE breast tumour blocks provide reliable sources of both germline and malignant DNA for investigation of genetic determinants of individual tumour responses to treatment
Source: Breast Cancer Res Treat. 2018 Apr 26;170(3):573–81. doi: 10.1007/s10549-018-4798-7 (PMC6022520; doi:10.1007/s10549-018-4798-7)
Supplement: Supplementary file 2 — Supplementary material 2 (DOCX 25 KB) [file 10549_2018_4798_MOESM2_ESM.docx]

**Table S1: ddPCR probes used to validate copy number alterations**

**Table S2: Shared and discrepant variants for exome sequencing**

| **Sample number** | **Total variants versus normal breast tissue** | **Total variants versus blood** | **Shared variants** | **Percentage of variants discrepant using normal breast or blood as reference^$^** | **Percentage of variants shared using normal breast or blood as reference*** |
| --- | --- | --- | --- | --- | --- |
| 1 | 29 | 30 | 27 | 15.63% | 84.38% |
| 2 | 321 | 341 | 320 | 6.43% | 93.57% |
| 3 | 12 | 11 | 10 | 23.08% | 76.92% |
| 4 | 85 | 90 | 84 | 7.69% | 92.31% |
| 5 | 55 | 59 | 55 | 6.78% | 93.22% |
|  |  |  |  |  |  |

**Table S3: Differences in number of mutations called with variation in VAF**

| **Sample id** | **Sample number** | **VAF** | **Total mutations versus normal** | **Total mutations versus blood** | **Shared mutations** | **Mutations unique to normal** | **Mutations unique to blood** |
| --- | --- | --- | --- | --- | --- | --- | --- |
| TF27 | 1 | 2% | 29 | 30 | 27 | 2 | 3 |
| TF27 | 1 | 5% | 25 | 27 | 24 | 1 | 3 |
| TF27 | 1 | 10% | 16 | 17 | 15 | 1 | 2 |
|  |  |  |  |  |  |  |  |
| TF29 | 2 | 2% | 321 | 341 | 320 | 1 | 21 |
| TF29 | 2 | 5% | 316 | 334 | 315 | 1 | 19 |
| TF29 | 2 | 10% | 303 | 319 | 302 | 1 | 17 |
|  |  |  |  |  |  |  |  |
| TF32 | 3 | 2% | 12 | 11 | 10 | 2 | 1 |
| TF32 | 3 | 5% | 11 | 10 | 9 | 2 | 1 |
| TF32 | 3 | 10% | 11 | 10 | 9 | 2 | 1 |
|  |  |  |  |  |  |  |  |
| TF43 | 4 | 2% | 85 | 90 | 84 | 1 | 6 |
| TF43 | 4 | 5% | 85 | 90 | 84 | 1 | 6 |
| TF43 | 4 | 10% | 67 | 68 | 66 | 1 | 2 |
|  |  |  |  |  |  |  |  |
| TF44 | 5 | 2% | 55 | 59 | 55 | 0 | 4 |
| TF44 | 5 | 5% | 53 | 57 | 53 | 0 | 4 |
| TF44 | 5 | 10% | 53 | 55 | 53 | 0 | 2 |
