# Supplementary material for: FFPE breast tumour blocks provide reliable sources of both germline and malignant DNA for investigation of genetic determinants of individual tumour responses to treatment
Source: Breast Cancer Res Treat. 2018 Apr 26;170(3):573–81. doi: 10.1007/s10549-018-4798-7 (PMC6022520; doi:10.1007/s10549-018-4798-7)
Supplement: Supplementary file 3 — Supplementary material 3 (DOCX 37 KB) [file 10549_2018_4798_MOESM3_ESM.docx]

**Table S6: Ampliseq gene list**

| **Gene name** | **Chromosome** | **Flanking genomic location start** | **Flanking genomic location end** |
| --- | --- | --- | --- |
| SHANK2 | chr11 | 70331530 | 70331532 |
| BEX1 | chrX | 102317869 | 102317871 |
| AIM2 | chr1 | 159033288 | 159033290 |
| C11orf71 | chr11 | 114270787 | 114270789 |
| CSMD3 | chr8 | 113650977 | 113650979 |
| GUK1 | chr1 | 228333287 | 228333289 |
| MDN1 | chr6 | 90388344 | 90388346 |
| MTNR1B | chr11 | 92714993 | 92714995 |
| SMC1A | chrX | 53432288 | 53432290 |
| ZNF407 | chr18 | 72346622 | 72346624 |
| ALPK2 | chr18 | 56246564 | 56246566 |
| FLII | chr17 | 18151156 | 18151158 |
| IKZF1 | chr7 | 50468105 | 50468107 |
| KCNJ12 | chr17 | 21318968 | 21318970 |
| PCTP | chr17 | 53848488 | 53848490 |
| DNAH17 | chr17 | 76511049 | 76511051 |
| HHEX | chr10 | 94449844 | 94449846 |
| HSD3B7 | chr16 | 30998296 | 30998298 |
| KDELR1 | chr19 | 48892874 | 48892876 |
| OTUD6A | chrX | 69283126 | 69283128 |
| PCIF1 | chr20 | 44569212 | 44569214 |
| PRRG3 | chrX | 150869157 | 150869159 |
| REXO4 | chr9 | 136279833 | 136279835 |
| SSC5D | chr19 | 56028988 | 56028990 |
| TTF2 | chr1 | 117617659 | 117617661 |
| TXNIP | chr1 | 145439651 | 145439653 |
| USP8 | chr15 | 50784949 | 50784951 |
| USP8 | chr15 | 50784954 | 50784956 |
| XCL1 | chr1 | 168549318 | 168549320 |
| ZSCAN4 | chr19 | 58189318 | 58189320 |
| DCAF4L1 | chr4 | 41984159 | 41984161 |
| HMGB2 | chr4 | 174254743 | 174254745 |
| HMGB2 | chr4 | 174254743 | 174254745 |
| POLD1 | chr19 | 50910373 | 50910375 |
| RANBP6 | chr9 | 6013636 | 6013638 |
| ZNF337 | chr20 | 25656579 | 25656581 |
| ARMC6 | chr19 | 19166112 | 19166114 |
| C15orf39 | chr15 | 75500156 | 75500158 |
| CYP8B1 | chr3 | 42917190 | 42917192 |
| DPY19L2 | chr12 | 64057542 | 64057544 |
| EFCAB4A | chr11 | 830322 | 830324 |
| EIF4G1 | chr3 | 184045690 | 184045692 |
| EXD3 | chr9 | 140289782 | 140289784 |
| FUT2 | chr19 | 49207033 | 49207035 |
| GAL3ST3 | chr11 | 65810837 | 65810839 |
| OTOP1 | chr4 | 4199269 | 4199271 |
| SEMA5A | chr5 | 9122827 | 9122829 |
| SORBS3 | chr8 | 22432366 | 22432368 |
| TFE3 | chrX | 48896696 | 48896698 |
| TRADD | chr16 | 67189154 | 67189156 |
| XPOT | chr12 | 64825379 | 64825381 |
| ABCA6 | chr17 | 67079141 | 67079143 |
| LRP5 | chr11 | 68157408 | 68157410 |
| MED16 | chr19 | 886162 | 886164 |
| PALD1 | chr10 | 72297631 | 72297633 |
| SMARCA2 | chr9 | 2054641 | 2054643 |
| TXNDC5 | chr6 | 7899880 | 7899882 |
| FAM136A | chr2 | 70524598 | 70524600 |
| USP35 | chr11 | 77921331 | 77921333 |
| XPOT | chr12 | 64825379 | 64825381 |
| ZIC3 | chrX | 136649704 | 136649706 |
| PMS2 | chr7 | 6037052 | 6037054 |
| RUNX1 | chr21 | 36252975 | 36252977 |
| TP53 | chr17 | 7578531 | 7578533 |
| PIK3CA | chr3 | 178952084 | 178952086 |
| TP53 | chr17 | 7577046 | 7577048 |
| CARD11 | chr7 | 2987417 | 2987419 |
| CBL | chr11 | 119146767 | 119146769 |
| CDKN2A | chr9 | 21994365 | 21994367 |
| CDKN2A | chr9 | 21994371 | 21994373 |
| CREBBP | chr16 | 3778082 | 3778084 |
| GNAS | chr20 | 57428721 | 57428723 |
| KIAA1598 | chr10 | 118666197 | 118666199 |
| PDGFRB | chr5 | 149502703 | 149502705 |
| PIK3CA | chr3 | 178936090 | 178936092 |
| TCF12 | chr15 | 57574755 | 57574757 |
| CARD11 | chr7 | 2962894 | 2962896 |
| KTN1 | chr14 | 56117105 | 56117107 |
| MEN1 | chr11 | 64577194 | 64577196 |
| RET | chr10 | 43610012 | 43610014 |
| BCR | chr22 | 23523575 | 23523577 |
| SF3B1 | chr2 | 198266775 | 198266777 |
| CTNNB1 | chr3 | 41275321 | 41275323 |
| MSH2 | chr2 | 47637367 | 47637369 |
| MBD6 | chr12 | 57919187 | 57919189 |
| GNAL | chr18 | 11753841 | 11753843 |
| IPO7 | chr11 | 9459366 | 9459368 |
| KIF13A | chr6 | 17790104 | 17790106 |
| KIF7 | chr15 | 90190207 | 90190209 |
| MAPK3 | chr16 | 30133152 | 30133154 |
| MAPKBP1 | chr15 | 42115284 | 42115286 |
| MCPH1 | chr8 | 6299592 | 6299594 |
| MMP25 | chr16 | 3100360 | 3100362 |
| MYH6 | chr14 | 23876299 | 23876301 |
| NPAS1 | chr19 | 47546128 | 47546130 |
| OPCML | chr11 | 132306657 | 132306659 |
| PIGN | chr18 | 59749911 | 59749913 |
| PSMG1 | chr21 | 40550496 | 40550498 |
| SGK3 | chr8 | 67710795 | 67710797 |
| TACSTD2 | chr1 | 59042497 | 59042499 |
| ZNF12 | chr7 | 6744801 | 6744803 |
| ANKRD36C | chr2 | 96616500 | 96616502 |
| SETDB1 | chr1 | 150933396 | 150933398 |
| VWA7 | chr6 | 31734428 | 31734430 |
| CA4 | chr17 | 58236605 | 58236607 |
| CENPF | chr1 | 214792013 | 214792015 |
| DNAAF2 | chr14 | 50100350 | 50100352 |
| DSP | chr6 | 7585260 | 7585262 |
| PCF11 | chr11 | 82878678 | 82878680 |
| PTCHD1 | chrX | 23411033 | 23411035 |
| RC3H2 | chr9 | 125642146 | 125642148 |
| SNX6 | chr14 | 35073371 | 35073373 |
| SYNPO2L | chr10 | 75415645 | 75415647 |
| ACTN1 | chr14 | 69369261 | 69369263 |
| CORO1A | chr16 | 30199272 | 30199274 |
| KIF4A | chrX | 69594102 | 69594104 |
| OR4C15 | chr11 | 55321917 | 55321919 |
| PKHD1L1 | chr8 | 110477136 | 110477138 |
| PPP1R15B | chr1 | 204375221 | 204375223 |
| SDHA | chr5 | 236602 | 236604 |
| BCAR1 | chr16 | 75276544 | 75276546 |
| PDE2A | chr11 | 72290580 | 72290582 |
| KRT33B | chr17 | 39521224 | 39521226 |
| CNTNAP2 | chr7 | 148112626 | 148112628 |
| CTR9 | chr11 | 10785694 | 10785696 |
| KIF26B | chr1 | 245850987 | 245850989 |
| RLTPR | chr16 | 67688539 | 67688541 |
| RP4-697K14.7 | chr20 | 62195064 | 62195066 |
| TSHZ3 | chr19 | 31770056 | 31770058 |
| ZNF551 | chr19 | 58198396 | 58198398 |
| ZNF764 | chr16 | 30566633 | 30566635 |
| ZNF780B | chr19 | 40541038 | 40541040 |
| ACRBP | chr12 | 6749634 | 6749636 |
| AL136218.1 | chr13 | 50008255 | 50008257 |
| CYP2A13 | chr19 | 41601806 | 41601808 |
| LGI3 | chr8 | 22006188 | 22006190 |
| OTOP1 | chr4 | 4199265 | 4199267 |
| ARMC4 | chr10 | 28250634 | 28250636 |
| B4GALT3 | chr1 | 161145667 | 161145669 |

**Table S7: Specialist Histopathologist and ASCAT estimates of tumour purity**

| **Sample number** | **Estimated tumour purity (%) by histopathologist** | **Estimated tumour purity (%) by ASCAT software** |
| --- | --- | --- |
| 1 | 60% | 52% |
| 2 | 60-70% | 63% |
| 3 | 70-80% | 84% |
| 4 | 80-90% | 67% |
| 5 | 70-80% | 66% |

**Table S8: Variation in mutations called using blood as germline with different stringency**

| **Sample** | **Number of Mutations** | **Min Tumour Coverage** | **Min Alt Reads** | **Min Tumour VAF** |
| --- | --- | --- | --- | --- |
| Sample 1 | 30 | 20 | 10 | 0.02 |
| Sample 1 | 30 | 15 | 10 | 0.02 |
| Sample 1 | 57 | 15 | 5 | 0.02 |
| Sample 1 | 57 | 10 | 5 | 0.02 |
| Sample 1 | 87 | 10 | 2 | 0.02 |
|  |  |  |  |  |
| Sample 2 | 11 | 20 | 10 | 0.02 |
| Sample 2 | 11 | 15 | 10 | 0.02 |
| Sample 2 | 44 | 15 | 5 | 0.02 |
| Sample 2 | 44 | 10 | 5 | 0.02 |
| Sample 2 | 79 | 10 | 2 | 0.02 |
|  |  |  |  |  |
| Sample 3 | 341 | 20 | 10 | 0.02 |
| Sample 3 | 341 | 15 | 10 | 0.02 |
| Sample 3 | 404 | 15 | 5 | 0.02 |
| Sample 3 | 404 | 10 | 5 | 0.02 |
| Sample 3 | 443 | 10 | 2 | 0.02 |
|  |  |  |  |  |
| Sample 4 | 90 | 20 | 10 | 0.02 |
| Sample 4 | 90 | 15 | 10 | 0.02 |
| Sample 4 | 124 | 15 | 5 | 0.02 |
| Sample 4 | 124 | 10 | 5 | 0.02 |
| Sample 4 | 135 | 10 | 2 | 0.02 |
|  |  |  |  |  |
| Sample 5 | 59 | 20 | 10 | 0.02 |
| Sample 5 | 59 | 15 | 10 | 0.02 |
| Sample 5 | 73 | 15 | 5 | 0.02 |
| Sample 5 | 73 | 10 | 5 | 0.02 |
| Sample 5 | 85 | 10 | 2 | 0.02 |

**Table S9: Breast cancer driver genes TCGA, Nik-Zainal, Pereira annotated**

| **Gene** | **Source** | **DOI** |
| --- | --- | --- |
| AKT1 | Nik-Zainal 2016 | doi:10.1038/nature17676 |
| AKT2 | Nik-Zainal 2016 | doi:10.1038/nature17676 |
| APC | Nik-Zainal 2016 | doi:10.1038/nature17676 |
| ARID1A | Nik-Zainal 2016 | doi:10.1038/nature17676 |
| ARID1B | Nik-Zainal 2016 | doi:10.1038/nature17676 |
| ASXL1 | Nik-Zainal 2016 | doi:10.1038/nature17676 |
| ATM | Nik-Zainal 2016 | doi:10.1038/nature17676 |
| ATR | Nik-Zainal 2016 | doi:10.1038/nature17676 |
| ATRX | Nik-Zainal 2016 | doi:10.1038/nature17676 |
| AXIN1 | Nik-Zainal 2016 | doi:10.1038/nature17676 |
| BCOR | Nik-Zainal 2016 | doi:10.1038/nature17676 |
| BRAF | Nik-Zainal 2016 | doi:10.1038/nature17676 |
| BRCA1 | Nik-Zainal 2016 | doi:10.1038/nature17676 |
| BRCA2 | Nik-Zainal 2016 | doi:10.1038/nature17676 |
| BUB1B | Nik-Zainal 2016 | doi:10.1038/nature17676 |
| CASP8 | Nik-Zainal 2016 | doi:10.1038/nature17676 |
| CBFB | Nik-Zainal 2016 | doi:10.1038/nature17676 |
| CBLB | Nik-Zainal 2016 | doi:10.1038/nature17676 |
| CCND1 | Nik-Zainal 2016 | doi:10.1038/nature17676 |
| CCND3 | Nik-Zainal 2016 | doi:10.1038/nature17676 |
| CCNE1 | Nik-Zainal 2016 | doi:10.1038/nature17676 |
| CDH1 | Nik-Zainal 2016 | doi:10.1038/nature17676 |
| CDK6 | Nik-Zainal 2016 | doi:10.1038/nature17676 |
| CDKN1B | Nik-Zainal 2016 | doi:10.1038/nature17676 |
| CDKN2A | Nik-Zainal 2016 | doi:10.1038/nature17676 |
| CDKN2B | Nik-Zainal 2016 | doi:10.1038/nature17676 |
| ZNF703 | Nik-Zainal 2016 | doi:10.1038/nature17676 |
| FGFR1 | Nik-Zainal 2016 | doi:10.1038/nature17676 |
| CIC | Nik-Zainal 2016 | doi:10.1038/nature17676 |
| CNOT3 | Nik-Zainal 2016 | doi:10.1038/nature17676 |
| CREBBP | Nik-Zainal 2016 | doi:10.1038/nature17676 |
| CTCF | Nik-Zainal 2016 | doi:10.1038/nature17676 |
| CUX1 | Nik-Zainal 2016 | doi:10.1038/nature17676 |
| DNMT3A | Nik-Zainal 2016 | doi:10.1038/nature17676 |
| ECT2L | Nik-Zainal 2016 | doi:10.1038/nature17676 |
| EGFR | Nik-Zainal 2016 | doi:10.1038/nature17676 |
| ERBB2 | Nik-Zainal 2016 | doi:10.1038/nature17676 |
| ERBB3 | Nik-Zainal 2016 | doi:10.1038/nature17676 |
| ERCC4 | Nik-Zainal 2016 | doi:10.1038/nature17676 |
| ESR1 | Nik-Zainal 2016 | doi:10.1038/nature17676 |
| FBXW7 | Nik-Zainal 2016 | doi:10.1038/nature17676 |
| FGFR2 | Nik-Zainal 2016 | doi:10.1038/nature17676 |
| FOXA1 | Nik-Zainal 2016 | doi:10.1038/nature17676 |
| FOXP1 | Nik-Zainal 2016 | doi:10.1038/nature17676 |
| GATA3 | Nik-Zainal 2016 | doi:10.1038/nature17676 |
| GNAS | Nik-Zainal 2016 | doi:10.1038/nature17676 |
| HRAS | Nik-Zainal 2016 | doi:10.1038/nature17676 |
| IGF1R | Nik-Zainal 2016 | doi:10.1038/nature17676 |
| KDM6A | Nik-Zainal 2016 | doi:10.1038/nature17676 |
| KRAS | Nik-Zainal 2016 | doi:10.1038/nature17676 |
| MAP2K4 | Nik-Zainal 2016 | doi:10.1038/nature17676 |
| MAP3K1 | Nik-Zainal 2016 | doi:10.1038/nature17676 |
| MDM2 | Nik-Zainal 2016 | doi:10.1038/nature17676 |
| MED23 | Nik-Zainal 2016 | doi:10.1038/nature17676 |
| MEN1 | Nik-Zainal 2016 | doi:10.1038/nature17676 |
| MLH1 | Nik-Zainal 2016 | doi:10.1038/nature17676 |
| MLL2 | Nik-Zainal 2016 | doi:10.1038/nature17676 |
| MLL3 | Nik-Zainal 2016 | doi:10.1038/nature17676 |
| MLLT4 | Nik-Zainal 2016 | doi:10.1038/nature17676 |
| MSH2 | Nik-Zainal 2016 | doi:10.1038/nature17676 |
| MYC | Nik-Zainal 2016 | doi:10.1038/nature17676 |
| NCOR1 | Nik-Zainal 2016 | doi:10.1038/nature17676 |
| NF1 | Nik-Zainal 2016 | doi:10.1038/nature17676 |
| NF2 | Nik-Zainal 2016 | doi:10.1038/nature17676 |
| NOTCH1 | Nik-Zainal 2016 | doi:10.1038/nature17676 |
| NOTCH2 | Nik-Zainal 2016 | doi:10.1038/nature17676 |
| NRAS | Nik-Zainal 2016 | doi:10.1038/nature17676 |
| PALB2 | Nik-Zainal 2016 | doi:10.1038/nature17676 |
| PBRM1 | Nik-Zainal 2016 | doi:10.1038/nature17676 |
| PDGFRA | Nik-Zainal 2016 | doi:10.1038/nature17676 |
| PHF6 | Nik-Zainal 2016 | doi:10.1038/nature17676 |
| PIK3CA | Nik-Zainal 2016 | doi:10.1038/nature17676 |
| PIK3R1 | Nik-Zainal 2016 | doi:10.1038/nature17676 |
| PMS2 | Nik-Zainal 2016 | doi:10.1038/nature17676 |
| PRDM1 | Nik-Zainal 2016 | doi:10.1038/nature17676 |
| PREX2 | Nik-Zainal 2016 | doi:10.1038/nature17676 |
| PTEN | Nik-Zainal 2016 | doi:10.1038/nature17676 |
| RB1 | Nik-Zainal 2016 | doi:10.1038/nature17676 |
| RHOA | Nik-Zainal 2016 | doi:10.1038/nature17676 |
| RUNX1 | Nik-Zainal 2016 | doi:10.1038/nature17676 |
| SETD2 | Nik-Zainal 2016 | doi:10.1038/nature17676 |
| SF3B1 | Nik-Zainal 2016 | doi:10.1038/nature17676 |
| SMAD4 | Nik-Zainal 2016 | doi:10.1038/nature17676 |
| SMARCA4 | Nik-Zainal 2016 | doi:10.1038/nature17676 |
| SPEN | Nik-Zainal 2016 | doi:10.1038/nature17676 |
| STAG2 | Nik-Zainal 2016 | doi:10.1038/nature17676 |
| STK11 | Nik-Zainal 2016 | doi:10.1038/nature17676 |
| TBX3 | Nik-Zainal 2016 | doi:10.1038/nature17676 |
| TET2 | Nik-Zainal 2016 | doi:10.1038/nature17676 |
| TP53 | Nik-Zainal 2016 | doi:10.1038/nature17676 |
| USP9X | Nik-Zainal 2016 | doi:10.1038/nature17676 |
| XBP1 | Nik-Zainal 2016 | doi:10.1038/nature17676 |
| ZFP36L1 | Nik-Zainal 2016 | doi:10.1038/nature17676 |
| ZNF217 | Nik-Zainal 2016 | doi:10.1038/nature17676 |
| AGTR2 | Pereira 2016 | doi:10.1038/ncomms11479 |
| BAP1 | Pereira 2016 | doi:10.1038/ncomms11479 |
| CHEK2 | Pereira 2016 | doi:10.1038/ncomms11479 |
| CTNNA1 | Pereira 2016 | doi:10.1038/ncomms11479 |
| FOXO3 | Pereira 2016 | doi:10.1038/ncomms11479 |
| GPS2 | Pereira 2016 | doi:10.1038/ncomms11479 |
| KMT2C | Pereira 2016 | doi:10.1038/ncomms11479 |
| TBL1XR1 | Pereira 2016 | doi:10.1038/ncomms11479 |
| AFF2 | TCGA 2012 | doi:10.1038/nature11412 |
| CLEC19A | TCGA 2012 | doi:10.1038/nature11412 |
| DCAF4L2 | TCGA 2012 | doi:10.1038/nature11412 |
| GPR32 | TCGA 2012 | doi:10.1038/nature11412 |
| GPS2 | TCGA 2012 | doi:10.1038/nature11412 |
| HIST1H2BC | TCGA 2012 | doi:10.1038/nature11412 |
| OR6A2 | TCGA 2012 | doi:10.1038/nature11412 |
| PTPN22 | TCGA 2012 | doi:10.1038/nature11412 |
| PTPRD | TCGA 2012 | doi:10.1038/nature11412 |
| RPGR | TCGA 2012 | doi:10.1038/nature11412 |
| RYR2 | TCGA 2012 | doi:10.1038/nature11412 |
| SEPT13 | TCGA 2012 | doi:10.1038/nature11412 |
| USH2A | TCGA 2012 | doi:10.1038/nature11412 |
